# Factors Influencing Running Performance During a Marathon: Breaking the 2-h Barrier

**DOI:** 10.3389/fcvm.2022.856875

**Published:** 2022-03-02

**Authors:** Elio Venturini, Francesco Giallauria

**Affiliations:** ^1^Cardiac Rehabilitation Unit, Department of Cardiology, Azienda USL Toscana Nord-Ovest, Cecina Civil Hospital, Livorno, Italy; ^2^Division of Internal Medicine and Cardiac Rehabilitation, Department of Translational Medical Sciences, Federico II University of Naples, Naples, Italy; ^3^Faculty of Sciences and Technology, University of New England, Armidale, NSW, Australia

**Keywords:** endurance, marathon performance, maximal oxygen uptake, running economy, advanced shoe technology

## Introduction

A few years ago, the brilliant view pointing of Joyner et al. (1) highlighted the physiological determinants of distance running performance, assuming that the barrier of the 2-h marathon would have been overcome in 2021–2022. October 14th 2019, Eliud Kipchoge broke this time barrier, concluding the marathon in 1:59:40. However the record was not homologated because not run under open marathon conditions and achieved with a rotation of 41 professional pacesetters. For the previous reasons, the current record is still 2:01:39, set in 2018 again by Kipchoge. The aim of this article is to update the key factors involved in the running performance.

## Subsections

The analysis starts from the physiology of the endurance race. Then it focuses on the impact on the performance of the environment and biomechanical factors, ending with individual factors such as nationality, genetics and sex.

## Discussion

### The Physiological Basis of the Marathon

The main determinants of the performance are three (2, 3): maximal oxygen uptake (VO_2_max), running economy (RE), i.e. the energy demand of running and the anaerobic threshold (AT). VO_2_max values in elite marathon runners are between 70 and 85 ml kg −1 min – 1 (1, 4) and running a marathon implies an average pace at 75–85% of VO_2_max (5). The VO_2_max is inversely correlated with the marathon time and predicts 59% of its variance (6). However, the absence of significant differences in VO_2_max between top class runners of different nationalities (7), the high level of average pace and the fact that the maximal oxygen uptake can vary (8), it shows that acting on this variable is not very useful for breaking the 2-h barrier.

The marathon performance correlates positively with the ability to maintain a high percentage of stroke volume and maximal cardiac output and, inversely, with the cost of running, that is the cardiac output/meter (9). Therefore, the improvement of the RE allows to run at a higher speed for the same oxygen uptake and for this reason is better predictor of performance than VO_2_max (7). Congenital or acquired factors can modify the RE. East African runners are characterized by the high fatty acid oxidation and slimmer lower legs. The reduced body mass with less heat build-up (detrimental for the performance) can have a positive impact on RE (10, 11). Also, the earlier exposure in life to high altitude and exercise can produce positive consequences on exercise capacity. In particular, a reduced arterial desaturation during exercise, an increased ventricular and muscle mass, differences in myosin light chain composition and motor coordination (10, 12).

On the other hand, a 10-week strength training, in addition to significantly increasing strength (24% for upper body lifting, 34% for lower body lifting), reduces RE by 4%, without modifying the VO_2_max (13). These data were confirmed in two meta-analyzes: even in highly trained runners, strength training in addition to improving maximum power and strength, reduces the energy cost of running (14, 15). Moreover, a greater hip flexor muscle strength relative to extensor muscle seems to be associated with a better RE, especially in male runners (16).

The RE is also affected by diet. Three weeks of intense training combined with a low-carb, high-fat ketogenic diet, in elite runners, despite an increase in peak aerobic capacity, impairs performance due to a reduction in RE (17). Indeed, if such a diet increases the ability of muscle to use fat as a substrate, keto-adaptation reduces muscle oxidation of carbohydrates and the ability to use glycogen, a limitation in high intensity endurance exercise (18). A chronic or periodised high carbohydrate availability, on the other hand, improves running performance, due to a reduced oxidative demand of the glycemic substrate (17).

Also the AT has a strong relationship with the level of performance in the marathon. The variable that has the highest correlation is the VO_2_max at least in elite male runners (19). In races of 10 km or more (but not over shorter distances), a significant correlation between running speed and AT has been demonstrated, especially in the group of older athletes (20). Therefore, training at the anaerobic threshold level induces a minimal increase in VO_2_max and in AT speed, but a significant increase in time to exhaustion at AT speed (21). This is probably due to better lactate clearance and improved performance.

Finally, age seemed to have a different impact on the determinants of performance. Due to the relationship with age, the maximum heart rate is reduced, so the highest values of maximum oxygen consumption, in elite marathoners, are reached around 27 years for males and 29 for females (22). However, the best performance is registered around the age of 35 due to the improvement in RE which offsets the drop in VO_2_max (23). Furthermore, the psychological profile of the elite runners (emotional stability, high motivation, strong psychic vigor) matures over the years and collaborates in improving performance later (24).

### Environmental and Biomechanical Factors

Topography influences the final time of the marathon. An analysis of the most famous marathon paths showed that the Berlin race (relatively flat with a final descent of about 15 km) has the fastest winning times (25). Climatic factors have a strong impact too, for example tail-wind and temperature. The first one improves the RE, but, when excessive it does not allow the homologation of the race time. Hyperthermia and dehydration reduce running speed and increase the risk of withdrawal. The best records of the marathon runs were achieved in spring or autumn, early in the morning and with an external temperature between 10 and 15°C (25). Recently it has been shown as airborne particulate matter (PM_10_) can settle in the airways of runners (26). The main marathons take place in large, generally highly polluted cities. The increased minute ventilation, induced by running, facilitates PM_10_ deposition. Air pollution can reduce the marathon performance, at least in women (every 10 μg m^−3^ in PM_10_, performance decrease by 1.4%) (27), as well as having a negative impact on health. To ensure “clean air” for elite runners, the PM content should be <5.5 μg m^−3^ (26).

Running in a sheltered position (drafting) is a way to improve performance especially as speed increases. The benefit is imputed to a reduced energy cost, but also to psychological reasons, especially not having to deal with the pace of the race (28). Drafting is easier to achieve as the number of athletes increases, i.e. in the first half of the marathon when selection has not yet begun. Having several runners to set the pace during the marathon could reduce the race time, even if the phenomenon is forbidden by the rules of the IAAF. However, keep a pace as uniform as possible, with very little speed changes along the race, allows the runner to be faster (25).

The availability since 2017 of the new advanced shoe technology (NAST) has led to an improvement in marathon performance. The athletes who ran with the NAST (29) significantly reduce the race time (−0.68%; *p* < 001), for different mechanisms. First the low weight of the shoes, with a reduction of oxygen consumption by 1% each change in the mass of the shoe of 100 gr (30). In addition, the introduction, in the sole, of carbon fibers plate, which when paired with a sole thickness of 40 mm is able to improve the RE by more than 4% (31). The structure of the sole is able to increase the energy return, due to the passive elastic recoil reducing the energy cost of the step. Furthermore, the greater stack-height of these shoes increases the lower-limb length improving the RE, especially in the case of long and thin legs with a higher moment of inertia and a reduced energy demand; it also increases stride length, a more efficient mechanism than stride frequency (31). If a curved stiff plate is inserted in the sole, this for the *teeter-totter* effect can return the energy of the runner, stored in the front part of the foot, as a force of reaction to the heel, during the take-off (30). It is also known that the involvement of the metatarsophalangeal joint increases with running speed, and that the foot dominates the stiffness of the foot-shoes and torque generation complex about the joint. By increasing stiffness, these shoes reduce the energy loss of the metatarsophalangeal joint (32). This explains why top-level athletes benefit most from NAST, as the RE improves as speed increases. A mathematical model estimated that with the use of NAST, the 2-h barrier will be overcome with a probability of more than 10% in 2025 (33).

### Nationality, Genetics and sex

Elite African runners, especially East African marathoners at the same career level, have faster marathon times than non-Africans. They also reach better results at a younger age and show a higher level of performance improvement, during their career (34, 35). The better performance of East African runners, in addition to the physiological mechanisms indicated, may depend on anthropometric factors (slender and long legs, resulting in lower internal work and high flexibility, short calcaneal tubers, long Achilles tendons, low body fat, high percentage of type I fibers) (36).

It is also difficult to define these characteristics as genetic. If the ACE I allele, associated with improved muscle and cardiovascular function during exercise, is overexpressed in Spanish runners (1), it has not been possible to define a single genetic profile in East African marathoners (37). The economic stimulus must also be added to this motivation: the prize in money is certainly a psychological incentive for athletes who come from countries with low per capita income.

At this point it is necessary to focus on the gender differences impacting on the performance. With the removal of the ban on participation in endurance races for women, there was a rapid improvement in marathon records, both for the increased number of runners in competitive races, and for the adoption of training techniques borrowed from the experience of males. However, due to greater muscle mass, heart size, higher hemoglobin concentration and lower body fat, the VO_2_max value in men is higher than in women (38); this is the physiological limit that explains the 10–12% slower times of women (39). Female runners have a higher AT than males and for the same level of performance, a lower AT speed. Nevertheless, they have a better RE, the factor highly correlated with AT speed (40). The superb performance of Paula Radcliffe (2:15:25), whose time is considered comparable to the men's marathon under 2 h, is due to a value of VO_2_max comparable to that of males, to an excellent RE, increased by training and to a higher speed of running (39). For 16 years it registered as world record and it is no coincidence that it was recently beaten by Brigid Kosgei (2:14:04), another Kenyan runner.

In conclusion, the main characteristics of the elite marathon runner (therefore with an optimal VO_2_max) able to break the barriers of 2 h, can be divided into physiological, environmental/biomechanical and individual. First of all, a better RE and a training, especially strength one, at the level of the AT; a diet with high carbohydrate availability can be helpful. A low airborne particulate matter, an optimal ambient temperature, a better pacing and drafting (according to IAAF rules) will be the ideal scenario to break the 2-h barrier. It is likely that the athlete will be East African around 35 years old. Last but not least, he will wear shoes with NAST that in recent years, has had the most important impact on the performance of runners,

## Author Contributions

EV contributed to the conception of the work and drafted the manuscript. EV and FG contributed to the acquisition, analysis, and interpretation of data for the work. FG critically revised the manuscript. All gave final approval and agree to be accountable for all aspects of work ensuring integrity and accuracy.

## Conflict of Interest

The authors declare that the research was conducted in the absence of any commercial or financial relationships that could be construed as a potential conflict of interest.

## Publisher's Note

All claims expressed in this article are solely those of the authors and do not necessarily represent those of their affiliated organizations, or those of the publisher, the editors and the reviewers. Any product that may be evaluated in this article, or claim that may be made by its manufacturer, is not guaranteed or endorsed by the publisher.
